# Editorial: Levothyroxine therapy in patients with hypothyroidism, volume II

**DOI:** 10.3389/fendo.2026.1940738

**Published:** 2026-07-30

**Authors:** Alessandro Antonelli, Paolo Miccoli

**Affiliations:** Department of Surgical, Medical, and Molecular Pathology and Critical Area, University of Pisa, Pisa, Italy

**Keywords:** cardiovascular effects of levothyroxine therapy, hypothyroidism, levothyroxine malabsorption, levothyroxine therapy, liquid levothyroxine, personalized levothyroxine therapy, softgel capsule levothyroxine

## 1 Introduction

Levothyroxine (L-T4) replacement therapy remains the standard care of treatment for primary, central, and postsurgical hypothyroidism (1). Initially, the therapeutic goal seems well-defined: restore thyroid hormone availability and achieve normalization of serum thyroid-stimulating hormone (TSH) levels. However, conventional weight-based dosing frequently fails to consistently achieve optimal targets, a considerable proportion of patients who are biochemically “euthyroid” continue to experience persistent symptoms, and the long-term effects on cardiovascular outcomes and bone mineral density (BMD) demand careful clinical management. Emerging evidence from studies published between 2024 and 2026 are driving a paradigm shift in endocrinology, moving the field beyond empirical, “one-size-fits-all” treatment strategies toward a more personalized approach to thyroid hormone replacement.

## 2 Overcoming gastrointestinal absorption barriers

The efficacy of oral L-T4 therapy depends on its bioavailability (60–80% in healthy adults) (2). Absorption occurs primarily in the jejunum and upper ileum and requires an acidic gastric environment for optimal tablet dissolution (3, 4).

### 2.1 Gastric pathologies and liquid formulations

A key advance in overcoming L-T4 absorption limitations has been the introduction of liquid formulations (5). A retrospective observational study by Fallahi et al. evaluated long-term TSH stability in hypothyroid patients with gastric disorders, including chronic gastritis, gastric cancer-related gastrectomy, and gastroplasty, comparing liquid L-T4 (n=84) with conventional tablet formulations (T-LT4; n=120). Proton pump inhibitors (PPIs) were commonly prescribed in both groups (66% in the tablet group and 51% in the liquid group), potentially affecting gastric pH and impairing tablet dissolution.

Although both formulations achieved similar TSH normalization after dose adjustment (5–9 months), longer follow-up (23–31 months) showed a clear difference between groups. At the final assessment, elevated TSH levels (>3.5 μIU/mL) were less frequent with liquid L-T4 than with tablets (5% vs 18%, p<0.05). These findings suggest that liquid L-T4, by bypassing gastric dissolution, provides greater long-term stability in patients with impaired gastric conditions, confirming also preceding published data (6, 7).

### 2.2 Lactose intolerance and excipient malabsorption

Beyond overt gastric disorders, biochemical incompatibilities between tablet excipients and the patient’s digestive capacity may also impair L−T4 efficacy. Lactose, a common excipient in conventional L−T4 tablets, makes lactose intolerance (LI)—a highly prevalent condition in adults—an often overlooked cause of refractory hypothyroidism (8–10).

In a literature review and case series, Ferrari et al. examined patients requiring high L−T4 doses (>1.7–2.0 μg/kg/day) to achieve biochemical euthyroidism. They showed that, when LI coexists with hypothyroidism, lactose-induced intestinal inflammation and altered transit impair L−T4 absorption. While a lactose-free diet may improve absorption, switching to a lactose-free liquid formulation effectively overcomes this limitation, normalizing TSH levels and maintaining long-term euthyroidism without restrictive dietary interventions.

### 2.3 Nutritional optimizations: the role of vitamin C

Co-administration of ascorbic acid may reduce gastric pH, thereby enhancing the dissolution of levothyroxine tablets. Agha et al. reported a high prevalence of vitamin C deficiency in hypothyroid patients requiring high-dose L-T4 therapy. In a subsequent randomized, double-blind trial, participants receiving 1 g of oral vitamin C daily alongside high-dose L-T4 for 16 weeks experienced a significant improvement in clinical symptoms, reflected by lower Zulewski clinical scores (p = 0.004), as well as a significant decrease in serum TSH levels (p = 0.043), suggesting enhanced L-T4 absorption.

## 3 Advanced algorithmic strategies for personalized dosing

Conventional weight-based dosing strategies (e.g., 1.6 μg/kg/day) may not provide an accurate estimate L-T4 requirements in overweight or obese individuals or in patients requiring TSH suppression after treatment for differentiated thyroid carcinoma (DTC).

### 3.1 Refining weight metrics

To improve dose estimation in patients with excess adiposity, Ma et al. investigated 385 thyroidectomized patients with DTC. The analysis revealed that although individuals with a higher body mass index (BMI) required greater total daily doses of L-T4 (p<0.001), the dose normalized to body weight was significantly lower than that of patients with a lower BMI (p<0.001). Regression analysis demonstrated that corrected body weight was the most reliable predictor of L-T4 requirements in patients with a normal BMI (≤23.9 kg/m²), whereas lean body mass provided the highest predictive accuracy in overweight and obese individuals (BMI ≥24.0 kg/m²). Compared with conventional empirical dosing, the optimized model substantially improved the accuracy of initial dose prediction, correctly estimating the required dose in 68.0% of cases versus 30.2% with the traditional approach (p<0.001).

### 3.2 Multi-factorial nomograms and machine learning

Zhang et al. identified biological sex, preoperative free thyroxine (FT4) levels, tumor stage (T), and BMI as independent determinants of postoperative TSH response. Based on these variables, the authors developed a predictive nomogram with a calibrated C-index of 0.757, offering a useful tool for the early identification of patients at risk of inadequate TSH suppression.

In parallel, machine learning approaches are increasingly supporting individualized L-T4 dosing. Liu et al. designed a support vector regression (SVR) model using six clinical parameters: body surface area, body weight, hemoglobin concentration, height, BMI, and age. Compared with conventional empirical dosing, the SVR-guided approach significantly increased the proportion of patients who achieved the target TSH level after the initial dose (52.09% vs. 10.53%) and reduced the mean time required to reach the therapeutic target from 115.50 to 62.61 days (p<0.05).

## 4 Long-term cardiovascular and bone safety

### 4.1 Echocardiographic dynamics

To investigate the effects of L-T4 on cardiac structure and function, Li performed a meta-analysis including six clinical studies. The pooled analysis showed no significant effect of L-T4 therapy on either left ventricular ejection fraction or the global myocardial performance index (LV Tei Index). In contrast, treatment produced significant improvements in parameters of left ventricular diastolic function, with both mitral E and mitral A velocities increasing significantly (p=0.0100). Further subgroup analysis indicated that a significant enhancement of the LV Tei Index was observed only after 12 months of continuous follow-up, suggesting that the beneficial effects of L-T4 become more evident over time and are mainly reflected in improved diastolic compliance and ventricular filling.

### 4.2 Quantifying cardiovascular risk

Rossi et al. highlighted that L-T4-induced subclinical hyperthyroidism is associated with a significantly increased risk of cardiovascular complications, including new-onset atrial fibrillation, left ventricular hypertrophy, and subclinical tachyarrhythmias. According to the authors, this increased risk may reflect the inability of exogenous L-T4 monotherapy to fully reproduce the physiological feedback mechanisms of endogenous thyroid hormone regulation.

Building on these observations, Pucci et al. evaluated the cardiovascular risk profile of 102 disease-free patients with DTC receiving long-term TSH-suppressive therapy by applying the 2021 European Society of Cardiology (ESC) risk assessment models, showing the need for dedicated cardio-oncology guidelines to balance effective cancer management with long-term cardiovascular risk.

### 4.3 Mitigating bone mineral loss

The long-term skeletal consequences of TSH-suppressive L-T4 therapy represent an important concern, as sustained reductions in TSH levels may promote excessive bone remodeling and shift the physiological balance toward increased osteoclastic activity. Patrizio et al. emphasized that the clinical impact of treating subclinical hypothyroidism (SCH) differs according to age, with potential benefits being more evident in younger individuals, whereas the effects on bone health in patients older than 65 years remain a matter of ongoing debate.

Further evidence was provided by Zhang et al., who prospectively evaluated 102 postmenopausal women with DTC and baseline osteopenia undergoing chronic TSH suppression. Their study compared conventional supplementation with calcium and vitamin D alone against a combined therapeutic strategy including technetium-99 methylene diphosphonate (99Tc-MDP). After 12 months, women receiving only calcium and vitamin D showed a significant deterioration in lumbar spine BMD, with values decreasing from 0.9148 ± 0.08 g/cm² to 0.8726 ± 0.08 g/cm² (p = 0.001). Conversely, the addition of 99Tc-MDP effectively prevented further bone loss (p = 0.035) and produced a significant reduction in the bone resorption marker β-CTX (p = 0.008), supporting its potential role in protecting skeletal health during prolonged TSH-suppressive treatment.

## 5 Deiodinase expression and the “dissatisfied patient”

Despite achieving adequate biochemical control, with normal serum TSH and FT4 levels during L-T4 monotherapy, some patients continue to experience fatigue, cognitive impairment, and weight gain, a condition commonly referred to as the “dissatisfied patient.”

### 5.1 Tissue-level heterogeneity and Hashimoto’s thyroiditis

The persistence of symptoms despite apparently adequate biochemical control may be partly explained by alterations in peripheral thyroid hormone metabolism, particularly involving type 2 deiodinase (DIO2), the enzyme responsible for the intracellular conversion of T4 into its active form, T3. Wu et al. investigated this mechanism in 162 patients with DTC following thyroidectomy. Among these patients, a subgroup exhibited an inadequate response to L-T4 therapy, characterized by the need for progressively higher doses despite insufficient TSH suppression. Immunohistochemical evaluation demonstrated significantly reduced local DIO2 protein expression in poor responders, which was associated with lower systemic FT3/FT4 ratios and impaired indices of central thyroid hormone sensitivity (p<0.05). These findings suggest that decreased tissue availability of DIO2 may contribute to a state of localized hypothyroidism at the peripheral level despite normal circulating hormone concentrations.

Consistent with this concept, Zhang et al. reported that conventional L-T4 monotherapy may not completely restore a physiological FT3/FT4 balance in euthyroid patients affected by Hashimoto’s thyroiditis (HT). Although the addition of liothyronine (L-T3) to L-T4 therapy represents a potential strategy for improving symptom control, complementary approaches, including selenium and vitamin D supplementation, may provide safer initial options for patients with persistent symptoms.

### 5.2 Subgroup classification

To better characterize the heterogeneous response observed among patients receiving thyroid hormone replacement, Welborn introduced a four-category framework aimed at distinguishing individuals who may genuinely benefit from T3 supplementation. This classification incorporates clinical features, including Woltman’s sign (delayed relaxation of deep tendon reflexes), as potential bedside markers for identifying true candidates for combination L-T4/L-T3 therapy:

1. T3-hypothyroidism: this category includes patients with reduced peripheral conversion of T4 into the active hormone T3, often related to impaired DIO2 activity. The presence of a positive Woltman’s sign may indicate a subgroup that could respond favorably to combined L-T4 and L-T3 treatment.
2. Anti-thyroid peroxidase (Anti-TPO) - Toxic Hypothyroidism: patients in this group present with systemic manifestations associated with elevated anti-TPO antibody levels. Significant improvements in quality of life have been reported following total thyroidectomy.
3. Hypothyroidism-in-Denial: this group refers to individuals with persistent symptoms that lack an identifiable biological explanation. In these cases, psychological support and cognitive-behavioral therapy represent the most appropriate therapeutic strategies.
4. Thyroxine-Sensitivity: a very uncommon condition characterized by immunologically mediated reactions to specific synthetic thyroxine preparations or to tablet excipients, such as coloring agents.

## 6 Technical interference and special populations

### 6.1 Unmasking macro-TSH

When laboratory findings are inconsistent with the clinical picture, immunoassay interference should be considered. Yin et al. applied polyethylene glycol (PEG) precipitation to identify high-molecular-weight serum complexes and found that macro-TSH, an inactive TSH-IgG autoantibody complex, was present in 36.6% of hypothyroid and 39.7% of subclinical hypothyroid samples. A TSH recovery threshold of 28% may help prevent unnecessary increases in L-T4 dosage and the risk of iatrogenic thyrotoxicosis.

### 6.2 End-stage renal disease

Zhao et al. highlighted that severe renal insufficiency alters thyroid hormone metabolism through uremic toxin accumulation and impaired hormone regulation. Consequently, L-T4 therapy requires individualized dosing, considering the altered pharmacokinetics and fluid changes associated with dialysis.

## 7 The new formulation landscape

Cappelli et al. highlighted the major advances in L-T4 therapy, from early thyroid extracts to modern oral formulations, while emphasizing that suboptimal treatment remains frequent. Gatta et al. showed that liquid L-T4 formulations can overcome absorption issues related to gastric pH alterations caused by drugs such as PPIs, calcium/iron supplements, and sevelamer, allowing more flexible co-administration.

Similarly, Virili et al. demonstrated that softgel capsules are less influenced by gastric acidity compared with traditional tablets, enabling a median 23% dose reduction in patients with impaired gastric acid secretion. Absorption is also affected by dietary factors, as Lai and Huang reported reduced L-T4 bioavailability associated with long-term tea and coffee consumption.

Finally, managing SCH in older adults requires age-specific reference ranges to avoid unnecessary treatment. Zhang et al. proposed a prospective multicenter study to validate these age-adjusted thresholds in patients over 60 years. This approach is essential, as Brenta et al. showed that reduced renal function is an independent predictor of SCH in older individuals at high cardiovascular risk, highlighting the need for accurate diagnosis to prevent systemic complications.

## 8 Conclusion: a blueprint for next-generation care

Recent evidence (2024–2026) supports a shift toward personalized levothyroxine therapy, with current strategies including:

- Optimizing levothyroxine dosing using individualized models based on machine learning algorithms and body composition.
- Improving drug absorption through liquid or softgel formulations and appropriate dietary management.
- Monitoring the cardiovascular and skeletal effects of long-term therapy, particularly during TSH suppression.
- Applying diagnostic techniques such as PEG precipitation to identify immunoassay interference caused by Macro-TSH when laboratory findings are inconsistent with the clinical presentation.

Finally, patients who remain symptomatic despite biochemical euthyroidism should be carefully evaluated. Altered peripheral thyroid hormone metabolism, including reduced type 2 deiodinase activity, may contribute to persistent symptoms in selected individuals, supporting consideration of individualized therapeutic strategies, including L-T4/L-T3 combination therapy when clinically appropriate. Overall, these advances may help bridge the gap between biochemical control and optimal clinical outcomes.
